# Effects of a Randomized Intervention to Improve Workplace Social Capital in Community Health Centers in China

**DOI:** 10.1371/journal.pone.0114924

**Published:** 2014-12-11

**Authors:** Xiaojie Sun, Nan Zhang, Kun Liu, Wen Li, Tuula Oksanen, Lizheng Shi

**Affiliations:** 1 Center for Health Management and Policy of Shandong University (key Lab of Health Economics and Policy, National Health and Planning Commission), Jinan, Shandong, China; 2 Health Bureau of Jinan, Jinan, Shandong, China; 3 Finnish Institute of Occupational Health, Helsinki, Finland; 4 Department of Global Health Systems and Development, School of Public Health and Tropical Medicine, Tulane University, New Orleans, Louisiana, United States of America; University of Geneva, Switzerland

## Abstract

**Objective:**

To examine whether workplace social capital improved after implementing a workplace social capital intervention in community health centers in China.

**Methods:**

This study was conducted in 20 community health centers of similar size in Jinan of China during 2012–2013. Using the stratified site randomization, 10 centers were randomized into the intervention group; one center was excluded due to leadership change in final analyses. The baseline survey including 447 staff (response rate: 93.1%) was conducted in 2012, and followed by a six-month workplace social capital intervention, including team building courses for directors of community health centers, voluntarily public services, group psychological consultation, and outdoor training. The follow-up survey in July 2013 was responded to by 390 staff members (response rate: 86.9%). Workplace social capital was assessed with the translated and culturally adapted scale, divided into vertical and horizontal dimensions. The facility-level intervention effects were based on all baseline (n = 427) and follow-up (n = 377) respondents, except for Weibei respondents. We conducted a bivariate Difference-in-Difference analysis to estimate the facility-level intervention effects.

**Results:**

No statistically significant intervention effects were observed at the center level; the intervention increased the facility-level workplace social capital, and its horizontal and vertical dimensions by 1.0 (p = 0.24), 0.4 (p = 0.46) and 0.8 (p = 0.16), respectively.

**Conclusions:**

The comprehensive intervention seemed to slightly improve workplace social capital in community health centers of urban China at the center level. High attrition rate limits any causal interpretation of the results. Further studies are warranted to test these findings.

## Introduction

A growing body of empirical research has suggested associations between social capital and health outcomes, including mortality [1], self-rated health [2], mental health [3]. According to the view of Putnam, social capital refers to those features of social relationships that facilitate collective action for mutual benefit [4]. In the workplace, social capital (WSC) refers to shared attitudes and values among members of a work unit, reciprocity, mutual respect and trust between workmates, collective action and participation in the networks at work, and trust in and trustworthiness of a supervisor [5]. Although previous studies have traditionally focused on social capital in residential or geographical areas, it has now been suggested that social capital at work should also be targeted [6], [7]. Workplace may constitute an important social unit because many people spend more working hours together than elsewhere, and workplace is a significant source of social relations. Compared to geographic units, workplaces might more appropriately capture the important social interactions and networks that constitute the core elements of social capital [8]. A recent review summarized the present extension of social capital research into workplaces [9]. WSC is contextually patterned, and workplace demographic and employment patterns as well as the size of the work unit are important in understanding variations in WSC [10], [11]. In a cohort of Finnish public sector employees, lower WSC was associated with the onset of depression [12], poorer health of employees [5], higher risk of co-occurrence of multiple lifestyle risk factors [13] and higher mortality [14]. Another group in Japan found that company-level mistrust was associated with higher likelihood of smoking and poor health among Japanese employees [15], [16], and individual perceptions of mistrust and lack of reciprocity at work had adverse effects on self-rated health among Japanese workers [16]. Recent studies in China also showed that higher individual-level WSC was associated with a lower likelihood of smoking among Chinese male employees [17], and WSC was positively associated with self-reported health and mental health [18]. Some researchers demonstrated that WSC represents a significant predictor of job satisfaction of those working in the field of patient care [19], [20]. Sometimes, WSC is also called organizational social capital (OSC). In a study focusing on Chinese universities and enterprises, OSC was associated with organizational commitment, and inversely associated with turnover intention [21].

WSC could play an important role in human resource management and performance improvement among working populations. Concern about turnover of medical staff is rising in a number of countries. While numerous factors have been linked to their turnover intention, job satisfaction and organizational commitment are regarded as the major risk factors [22]–[24]. Therefore, improving job satisfaction and organizational commitment are important management strategies of human resources for health institutions. Ling et al. put forward five types of organizational commitment among Chinese working population: affective, normative, ideal, economic and choice [25]. They analyzed determinants of these types, and found such organizational or workplace factors as trust toward the leaders, organizational support, the maintenance behavior of the leader, the perceived dependability of the organization, employee's satisfaction towards colleagues, and the morale of employee's belonging organization. In fact, several of these factors can be integrated into the theoretical framework of WSC.

How to attract and retain qualified human resources is a major challenge in the recent years, for governments at all levels in China to develop an effective and efficient community health services, especially after a great deal of money has been invested in the infrastructure of community health services. A survey in Beijing in 2009 showed that 36.9% of community health staff had turnover intentions [26]. A review [27] indicated that main influencing factors of job satisfaction included income level, work environment, facility management level and occupation development. In the new health reform of China, strengthening community health services is a key point. Community health workers in China are bearing a great deal of job pressures while receiving poor salary and welfare, which may exacerbate low job satisfaction and high turnover rate. It has been suggested that fostering social capital among workers might increase organizational commitment and decrease turnover [28]. However, only a limited number of empirical intervention studies have sought to foster social capital in community settings. A cluster randomized intervention study in rural South Africa found higher levels of structural and cognitive social capital in the intervention group than the comparison group [29]. In Japan, the Taketoyo Project aims at promoting social interaction among community-dwelling older adults in several communities [30]. Murayama et al. argued that observed increase of social support and participation in the Project improved health [30]. However, it is not known how to increase social capital in the workplace. Therefore, we aimed to examine whether WSC will be improved after we implemented a WSC intervention in community health centers (CHCs) in China.

## Methods

### Ethics statement

The Institutional Review Board (IRB) of School of Public Health in Shandong University of China approved the study. The IRB approved the consent procedure, and all participants provided their written informed consent to participate in this study.

### Study design

#### Study setting

**Figure 1:**
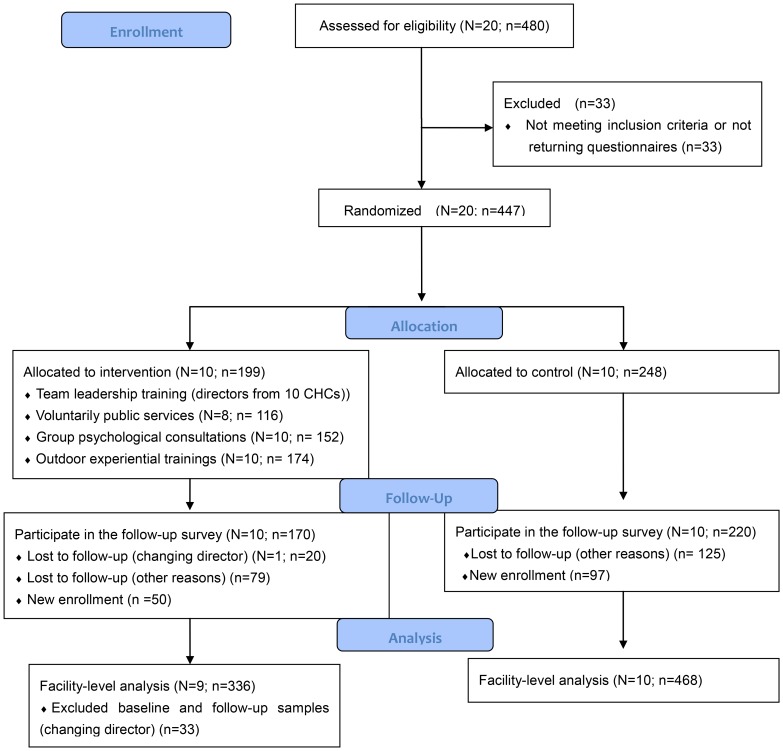
The flowchart of this study. This figure shows the study design of the study. N is the number of CHCs, and n is the number of staff in selected CHCs. In baseline survey, 480 questionnaires were distributed, and we finally got 447 valid questionnaires returned by eligible respondents. And then, 10 centers were randomly selected as the intervention group. The numbers of involved intervention centers and staff in each activity are shown in the figure. 390 staff participated in the follow-up survey, and the numbers of lost to follow-up and new enrollment are also shown. Other reasons for lost to follow-up included retirement, turnover, sick leave, causal leave, refusing to fill in the follow-up questionnaires, and uncompleted follow-up WSC answers. Finally, the facility-level intervention effects were evaluated based on all baseline and follow-up samples (n = 336+468 = 804) except Weibei respondents (n = 33).

This study was conducted in 2012–2013 in Jinan of Shandong province, located in China's eastern coastal area and one of China's more economically developed regions (a per capita GDP in 2012 of CNY 51,897, about $US 8,257). Jinan, the Capital city in Shandong province, is one of the Chinese cities which initiated community health services very early. By the end of 2012, there were 60 CHCs in five urban districts. Based on power calculations referring to the pilot test of this intervention program, at least 20 centers (10 intervention centers and 10 control centers) were needed. According to the geographical distribution, willingness in participation, and facility characteristics, we selected 20 middle-size centers (the number of employees ranged from 13 to 56, and in 75% of the centers it was between 15 and 32; 2 from Licheng, 4 from Lixia, 2 from Huaiyin, 6 from Tianqiao and 6 from Shizhong) to participate in this study. Those centers, which were too large or small, were excluded. Fig. 1 shows the flowchart of this study.

#### Randomization

We used the stratified site randomization to randomize the 20 centers into the intervention and control groups. Firstly, we assigned the first random digit generated by a computer to each CHC, and then ranked them in an ascending order. Secondly, we assigned the second random digit to each CHC, and then ranked CHCs within each districts in an ascending order. Finally, those in even positions within each district were distributed into the intervention group, and the rest were distributed into the control group.

#### Procedures

1. In December 2012, the baseline survey was responded by 447 staff members from 20 centers (response rate: 93.1%). In each center, except for the director, those whose job tenures were less than 6 months, and temporary staff, all other employees were asked to anonymously fill in a questionnaire inquiring demographic and job related information, WSC, job satisfaction, organizational commitment, and turnover intention. Basic institutional information was given by the contact person of each center.
2. According to the pilot experiences and literature review [31]–[34], we made a WSC intervention package (January to June, 2013) including four activities, which had been suggested to promote certain aspects of WSC. One was the team leadership training activity, which was a one-day team building courses for CHC directors; the other three non-leadership activities for CHC staff included self-organizing voluntarily public services for disadvantaged community residents, a half-day group psychological consultation and one-day outdoor experiential trainings. The directors in intervention centers were asked to join and coordinate all activities. The team building courses including team management and communication skills and practical team leadership experiences were given in January. The trainers included a team management expert, the director of health bureau in one district of Jinan, a psychology expert and an excellent CHC director famous for team building. To improve the sense of group solidarity and communications among staff, each intervention center was asked to self-organize public services for the older adults, the disabled or the poor within their communities during March to April. Two psychology experts supplied half-day consultations for each center during April to May, focusing on team communications and stress management. Finally, one-day outdoor trainings aiming at improving team coordination and communications were organized in June.
3. The follow-up survey was done in July, 2013 and similar information to the baseline survey was collected anonymously. Altogether 390 staff members from 20 centers returned valid questionnaires (response rate: 87.9%). Of baseline respondents, 223 employees completely responded to the follow-up survey (response rate 49.9%).

#### Outcomes Measure

WSC was assessed by the translated and culturally adapted 8-item measure developed in the Finnish Public Sector study [35]. It has been shown to be a reliable and valid measure of social capital [35]. A recent psychometric evaluation in Chinese employees has demonstrated the scale to have high internal consistency (Cronbach's alpha of 0.94) [18]. In our study, the Cronbach's alpha coefficients of total scale, horizontal and vertical subscales were 0.90, 0.85 and 0.87, respectively. The response options were given in a 5-point Likert scale. We computed the WSC total score (ranging from 8 to 40) by summing scores of all the 8 items, and higher score indicated higher social capital. According to the factor analysis results in our study, following the dimensions in previous studies [36], [37], we divided the eight items into two dimensions: vertical WSC and horizontal WSC (see table 1). The vertical dimension is related to employees' relations with their employers and supervisors, while the horizontal dimension captures issues related to bonding and bridging social capital [37]–[39], which involves social contacts, cooperation and trust in relation to coworkers. We computed the score of each dimension by summing the scores of all the items in each dimension. The average scores of individual WSC total score, vertical WSC score and horizontal score within each center were computed to represent the facility-level WSC.

**Table 1:** Workplace social capital (WSC) dimensions and indicators.

Dimension	Indicators
**Vertical WSC**	We can trust our supervisor.
	Our supervisor treats us with kindness and consideration.
	Our supervisor shows concern for our rights as an employee.
**Horizontal WSC**	We have a ‘we are together’ attitude.
	People feel understood and accepted by each other.
	People in the work unit cooperate in order to help develop and apply new ideas.
	Do members of the work unit build on each other's ideas in order to achieve the best possible outcome?
	People keep each other informed about work-related issues in the work unit.

#### Other variables

The socio-demographic variables were sex, age group (less than or equal to 29, 30–39, 40–49, 50 and above) and education level (high school and below, some college/associate degree, bachelor and above). The job characteristics were occupation type (doctors, nurses and others), professional title (no, entry-level, middle and above (middle, associate senior and senior)), income gap group (RMB 1500 and below, RMB 1501–2499, RMB2500 and above), and weekly working hours (40 and below, >40). Here the “income gap” means the difference between the expected income and the actual income. The health center level characteristics were the proportion of permanent staff, the average age of staff, operation years, and the number of staff.

### Statistical analysis

Descriptive statistics were used to describe and compare the mean values of facility-level characteristics between the intervention and control groups. Two-sample Wilcoxon rank-sum (Mann-Whitney) test was used to compare operation years of CHC which were non-normally distributed. Pearson Chi-square tests were used to describe and compare the proportions for individual-level variables. Welch's t test was used when two samples had unequal variances.

The social capital theory posits that social capital is both an individual and group-level phenomenon. In this study, we focused on the health center level analysis, and analyzed the intervention effects in all baseline and follow-up participants, except those from Weibei Center, leading to a sample of 19 centers, 427 baseline respondents and 377 follow-up respondents. Because the director of Weibei center (an intervention center) changed during the intervention, this center was removed from the analysis. Olsen et al., argued that fundamental changes at worksites need to be taken into account when planning intervention studies [40].

A significant proportion of baseline respondents did not respond to the follow-up study. To address potential sample attrition bias due to non-response, we described and compared the covariates and outcomes between the remaining participants (participating in both of baseline and follow-up surveys) and those lost to follow-up in the intervention and control groups [41].

We conducted a bivariate difference-in-differences (DID) analysis using paired T-test to analyze the facility-level WSC intervention effects. The DID method compares the differences in WSC in pre- and post-intervention periods in the intervention and control groups. The DID approach seeks to measure the effect of the intervention while controlling for unobserved heterogeneity. The method relies on the assumption of a “parallel trend” which states that time trends for the outcome would have been identical in the intervention and the control group in the absence of the intervention. This assumption is strong yet never testable. Moreover, the DID estimates are more reliable when the outcomes are compared just before and just after the intervention, as done in our study, because the parallel trends assumption is more likely to hold over a short time window.

Stata12.0 software was used to analyze the data with the two-tailed significance level at 0.05.

## Results

Table 2 shows the success of the randomization at the facility level in the baseline characteristics. There were no statistically significant differences in any of variables, except for the professional title. In the intervention group, the proportion of entry-level professionals was higher and that of middle and above level lower than in the control group. The composition of the groups also differed slightly in relation to occupation type (p = 0.07) and income gap group (p = 0.10).

**Table 2:** Baseline characteristics comparison between the intervention and control CHCs.

	Intervention centers (N = 10)	Control centers (N = 10)	P value
**Facility characteristics**			
** % of permanent staff (Mean(Median))**	48.2 (40.8)	51.4 (57.9)	0.82
**Average age of staff (Mean(Median))**	35.3 (35.3)	36.2 (36.1)	0.56
** Operation years of the Community health center (Mean(Median))**	9.2 (9.5)	8 (6)	0.61
**Number of staff (Mean(Median))**	23.3 (23.5)	27.1 (21)	0.47
**Individual characteristics**			
**Gender (%)**			0.61
** Male**	15.1	17.3	
**Age group(%)**			0.25
** < = 29**	40.7	28.3	
** 30∼**	22.1	31.2	
** 40∼**	25.1	28.3	
** > = 50**	12.1	12.2	
**Education level(%)**			0.34
** High school and below**	17.2	18.2	
** Some college/associate**	50.5	43.7	
** Bachelor and above**	32.3	38.1	
**Occupation type (%)**			0.07
** Doctors**	28.6	39	
** Nurses**	41.2	34.1	
** Others**	30.2	26.8	
**Professional title (%)**			0.02
** no**	24	18.9	
** Entry-level**	43.9	35.4	
** Middle and above**	32.1	45.7	
**Income gap group(%)** *			0.10
** < = 1500**	42.7	40	
** 1501-**	37.3	31.3	
** > = 2500**	20	28.8	
**Weekly work hours (%)**			0.63
** >40**	35.7	37.9	
**WSC total score (Mean(SD))**	31.5 (2.83)	31.4(1.8)	0.92
**Horizontal WSC (Mean(SD))**	19.7(1.44)	19.5(1.19)	0.73
**Vertical WSC (Mean(SD))**	11.8(1.42)	11.9(0.73)	0.85
			

*Here, the income cap means the difference between the expected monthly income and actual monthly income.

* Welch's t test was used when two samples had unequal variances.

Table 3 presents baseline characteristics for the remaining participants who participated in both baseline and follow-up surveys, and those who were lost to follow-up. The study had no differences in attrition rates between the intervention and control arms. Except weekly work hours and WSC scores, there were no statistically significant differences of baseline characteristics between the remaining participants and those lost to follow-up in both groups. In the control group, the percent with more than 40 weekly work hours among those lost to follow-up was 12.3% higher (p = 0.046) than that among the remaining participants. In the intervention group, the scores of WSC total scale, horizontal and vertical dimensions among those lost to follow-up were respectively 2.9, 1.9 and 0.9 points higher than the remaining participants (all p<0.01).

**Table 3:** Baseline characteristics of the remaining participants, and those lost to follow-up.

	Remaining participants		Participants lost to follow-up			
	Intervention group (1) (N = 100)	Control group(2) (N = 123)	Intervention group (3) (N = 99)	Control group(4) (N = 125)	P value (1 vs 3)	P value (2 vs 4)
**Gender**					0.12	0.66
** Male**	19(19)	20(16.3)	11(11.1)	23(18.4)		
**Age group**					0.11	0.47
** < = 29**	40(40)	37(30.3)	41(41.4)	33(26.4)		
** 30∼**	28(28)	35(28.7)	16(16.2)	42(33.6)		
** 40∼**	24(24)	38(31.2)	26(26.3)	32(25.6)		
** > = 50**	8(8)	12(9.8)	16(16.2)	18(14.4)		
**Education level**					0.92	0.18
** High school and below**	16(16.6)	28(22.8)	18(18.2)	17(13.7)		
** Some college/associate**	51(51.5)	50(40.7)	49(49.5)	58(46.8)		
** Bachelor and above**	32(32.3)	45(36.6)	32(32.3)	49(39.5)		
**Occupation type**					0.5	0.11
** Doctors**	28(28)	40(32.8)	29(29.3)	56(45.2)		
** Nurses**	45(45)	48(39.3)	37(37.4)	36(29)		
** Others**	27(27)	34(27.9)	33(33.3)	32(25.8)		
**Professional title**					0.69	0.56
** no**	22(22.5)	26(21.7)	25(25.5)	20(16.3)		
** Entry-level**	46(46.9)	41(34.2)	40(40.8)	45(36.6)		
** Middle and above**	30(30.6)	53(44.2)	33(33.7)	58(47.2)		
**Income gap group** *					0.14	0.58
** < = 1500**	36(37.9)	49(41.9)	43(47.8)	47(38.2)		
** 1501-**	42(44.2)	38(32.5)	27(30)	37(30.1)		
** > = 2500**	17(17.9)	30(25.6)	20(22.2)	39(31.7)		
**Weekly work hours**					0.84	0.046
** >40**	35(35)	39(31.7)	36(36.4)	55(44)		
**WSC total score (SD)**	30.1(4.66)	31.4(4)	33(5.55)	31.5(4.87)	<0.001	0.91
**Horizontal WSC (SD)**	18.7(3.13)	19.6(2.65)	20.6(3.26)	19.5(3.06)	<0.001	0.97
**Vertical WSC(SD)**	11.4(2.09)	11.9(1.82)	12.3(2.67)	11.9(2.31)	0.005	0.78

*Except three WSC indicators, the numbers inside and outside the brackets were sample size and composition %, and these percents were computed after deleting records with missing information.

*Here, the income cap means the difference between the expected monthly income and actual monthly income.

* Welch's t test was used when two samples had unequal variances.

**Figure 2:**
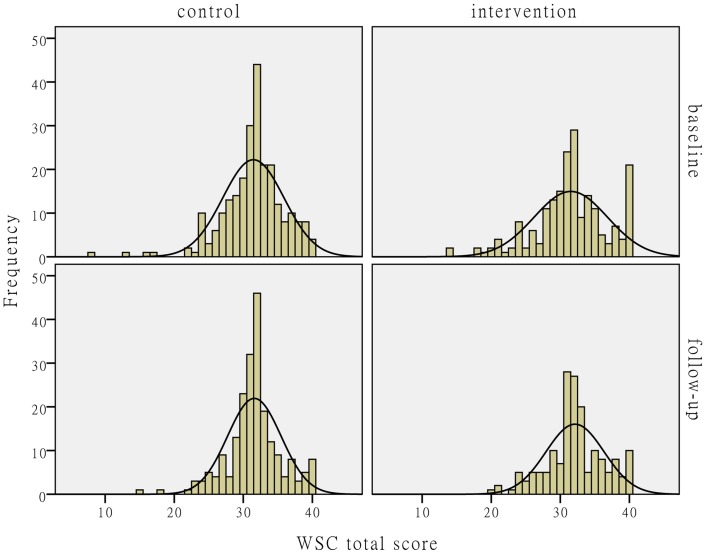
The distribution comparison of individual-level WSC total score. Fig. 2 shows the distributions of individual-level WSC total score. The histograms and fitting normal distribution curves in the upper-left and lower-left corners in the figure represent the observation frequencies and distributions before and after the intervention in the control group. The histograms and fitting normal distribution curves in the upper-right and lower-right corners in the figure represent the observation frequencies and distributions before and after the intervention in the intervention group.

**Figure 3:**
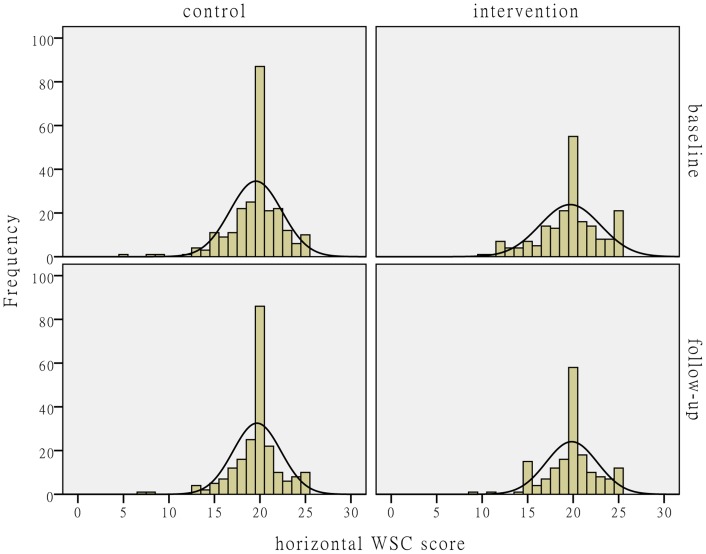
The distribution comparison of individual-level horizontal WSC score. Fig. 3 shows the distributions of individual-level horizontal WSC score. The histograms and fitting normal distribution curves in the upper-left and lower-left corners in the figure represent the observation frequencies and distributions before and after the intervention in the control group. The histograms and fitting normal distribution curves in the upper-right and lower-right corners in the figure represent the observation frequencies and distributions before and after the intervention in the intervention group.

**Figure 4:**
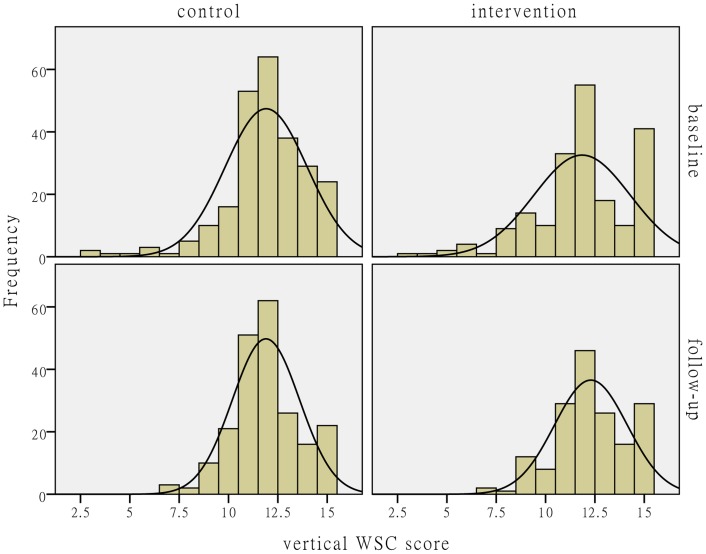
The distribution comparison of individual-level vertical WSC score. Fig. 4 shows the distributions of individual-level vertical WSC score. The histograms and fitting normal distribution curves in the upper-left and lower-left corners in the figure represent the observation frequencies and distributions before and after the intervention in the control group. The histograms and fitting normal distribution curves in the upper-right and lower-right corners in the figure represent the observation frequencies and distributions before and after the intervention in the intervention group.

Figs. 2–4 show the distributions of the individual-level WSC and its horizontal and vertical dimensions. The figures show that the WSC variables in the control group hardly changed after the intervention. The mean values of the social capital variables in the intervention group slightly increased with reduced variances.

Table 4 present the results from the bivariate DID analysis on the effects of the WSC intervention. After the intervention, the facility-level WSC total score, horizontal WSC score and vertical WSC score in the intervention group increased by 1.2, 0.5 and 0.8 points. The same variables hardly changes in the control group. The DID estimators showed that the intervention increased the facility-level WSC total score, horizontal WSC score and vertical WSC score by 1.0, 0.4, and 0.8 points. None of these changes were statistically significant (table 4).

**Table 4:** Facility-level WSC DID analysis results.

Dependent variables	Baseline	Follow-up	Difference 1	DID
**WSC total score (SE)**				
**Intervention**	N = 9	N = 9		
	31.1(0.87)	32.3(0.81)	1.2 (0.85) (p = 0.17)	
**Control**	N = 10	N = 10		
	31.4(0.57)	31.6(0.42)	0.2 (0.31) (p = 0.61)	
**Difference 2**	−0.3(1.02)	0.7(0.89)		1.0 (0.9)
	(p = 0.75)	(p = 0.38)		(p = 0.24)
**Horizontal WSC score (SE)**				
**Intervention**	N = 9	N = 9		
	19.4(0.43)	19.9(0.50)	0.5 (0.36) (p = 0.21)	
**Control**	N = 10	N = 10		
	19.5(0.37)	19.6(0.27)	0.1(0.27) (p = 0.57)	
**Difference 2**	−0.1(0.57)	0.3(0.56)		0.4 (0.45)
	(p = 0.94)	(p = 0.6)		(p = 0.46)
**Vertical WSC score (SE)**				
**Intervention**	N = 9	N = 9		
	11.6(0.46)	12.4(0.33)	0.8(0.51) (p = 0.16)	
**Control**	N = 10	N = 10		
	11.9(0.23)	11.9(0.23)	0(0.09) (p = 0.9)	
**Difference 2**	−0.3(0.49)	0.5(0.4)		0.8(0.52)
	(p = 0.57)	(p = 0.21)		(p = 0.16)

* Welch's t test was used when two samples had unequal variances. Paired t test was only used to test the differences between the baseline and follow-up WSC scores.

*The values in the brackets are SEs.

*Difference1 =  Follow-up – Baseline; Difference 2 =  intervention – control; DID =  Difference 1 (intervention)-Difference 1 (control).

## Discussions

This is the first study to suggest that workplace social capital can be improved using a 6-month comprehensive intervention package. The intervention package was comprised of four parts: the team building course for CHC directors, self-organized volunteer public services for disadvantaged community residents, group psychological consultations and outdoor trainings. Compared to the pre-intervention situation, the total scores of workplace social capital and its vertical and horizontal dimensions were improved after the intervention in Chinese community health centers. The increase was observed in both the individual-level and facility-level workplace social capital, although the facility-level increase was smaller and insignificant. However, high attrition rates limit any causal interpretation of the results. Further studies are needed to test these findings.

The leader has the responsibility to plan, coordinate, and monitor the group's activities and to convey a vision, inspiring team collaboration [42]. Therefore, the involvement and commitment of the community health center directors was essential in the study. If a CHC director did not understand the significance and skills of team leadership and communications, the director would not actively and effectively prioritize and participate in the WSC intervention. So, the reason that we put the team-building course for CHC leaders in the beginning the whole intervention is not only to directly promote the vertical WSC focusing on the vertical relationship between CHC directors and staff, but also to maximize the involvement and commitment of the CHC directors. The increased WSC scores together with decreased variations of the total scale and the vertical dimension in the intervention group gives support that our strategy was necessary and effective. Similarly, a previous study, focusing on improving group cohesion and nursing satisfaction, found that there is a critical role of nurse managers, and they need to be committed to the development of the leadership skills and team cohesiveness [31]. In China, the majority of CHC directors who originally were doctors or nurses lack in formal team management training and skills. Indeed, it has also been emphasized in the United States that additional leadership training opportunities for active and future CHC medical directors are needed [43].

Among three non-leadership intervention activities focusing on community health staff, human resource management literature has suggested that voluntarily public service programs are not only linked with the enhancement of the organization's public image [44]–[46], but also associated with higher organizational commitment [32]. It is plausible that, providing voluntarily public services for disadvantaged community residents by CHC staff members can effectively improve team cohesiveness through establishing a good public image and improving organizational commitment. Psychological consultations were included in the intervention because needs assessment indicated that CHC staff was lack of skills of communicating with colleagues and directors, and dealing with job stress. The positive impact of such psychological consultation activities was found on communication and job satisfaction of nursing staff [33]. Outdoor experiential trainings have been extensively used in team building programs [47]. Results from an outdoor training study on team-building for health care in Italy, showed a good levels of satisfaction and knowledge increase, which resulted in better team-building and mutual appreciation [34].

### Limitations

This study has six main limitations. Firstly, owing to the relatively small sample size, it was impossible to examine the separate effect of each intervention activity on WSC improvement. Secondly, the follow-up survey was conducted right after the 6-month intervention. Thus, we had no data available to observe any long-term effects of the WSC intervention. Thirdly, owing to the small sample sizes of health centers, the randomization may not necessarily have guaranteed the high comparability between the intervention and the control groups. Fourthly, our study had a relatively high proportion of lost to follow-up, which may have introduced attrition bias. Such bias is often assumed to exaggerate the effect. In addition, the attrition rate limits any causal interpretation of the results. Fifth, the baseline WSC scores were higher in the participants lost to follow-up, which may have lead to an overestimation of the facility-level intervention effects especially when concerning the possibility of regression to the mean. Finally, this intervention was implemented in the context of urban CHCs in a single city, and, therefore, the results are not necessarily generalizable to other health facilities (such as hospitals) or rural areas in China or other countries.

### Conclusions

The findings of this study suggest that implementing a comprehensive WSC intervention slightly improved WSC in community health centers of urban China. However, the high drop-out rate in the study limits any causal interpretation of the results. Further studies are needed to test these findings, and especially, to examine whether an increase in workplace social capital in community health centers can increase organizational commitment and decrease turnover rates among staff.

## Supporting Information

S1 Database **Baseline individual characteristics in the intervention and control centers.** This database includes the individual characteristics data of baseline participants in the intervention and control centers, which were used to compare the differences of the individual characteristics between the intervention and control centers, shown in table 2. And the data were also used to compare the differences of baseline characteristics between the remaining participants and those lost to follow-up, shown in table 3.
(DTA)

S2 Database **Baseline facility characteristics in the intervention and control centers.** This database includes the facility characteristics data of the intervention and control centers, which were used to compare the differences of the facility characteristics between the intervention and control centers, shown in table 2.
(DTA)

S3 Database **Individual-level and facility-level WSC scores before and after the intervention.** This database includes the individual-level WSC total score, horizontal and vertical WSC scores among all the baseline and follow-up participants in the intervention and control centers, and the aggregated facility-level WSC total score, horizontal and vertical WSC scores, which were used to produce Figs. 2-4 and the facility WSC DID analysis results in table 4.
(DTA)
